# The vascular glycocalyx is not a mechanosensor in conduit arteries in the anesthetized pig

**DOI:** 10.7717/peerj.8725

**Published:** 2020-03-02

**Authors:** Therese Ruane-O’Hora, Ahmad Ahmeda, Farouk Markos

**Affiliations:** 1 Department of Physiology, School of Medicine, University College Cork, Cork, Ireland; 2 College of Medicine, QU Health, Qatar University, Doha, Qatar

**Keywords:** Hyaluronic acid, Glycocalyx, Flow mediated dilation, fMLP, Neuraminidase, Heparan sulfate

## Abstract

**Background:**

The role of the glycocalyx as the endothelial sensor of an increase in blood flow was assessed in the iliac artery in vivo.

**Methods:**

Acetylcholine-induced flow mediated dilation was evaluated before and after vascular glycocalyx disruption. This was accomplished by exposing the iliac lumen to the chemotactic agent fMLP (1 μM; *n* = 6 pigs), concomitant heparinase III (100 mU ml^−1^) and hyaluronidase (14 mg ml^−1^) (*n* = 4), and neuraminidase (140 mU ml^−1^; *n* = 5), for 20 min in separate iliac artery preparations. Only one lumen intervention per iliac was conducted.

**Results:**

For the heparinase III + hyaluronidase experiment, the iliac diameter increased by an average of 0.54 ± 0.11 mm before and 0.45 ± 0.03 mm after the enzymes (*P* = 0.42; paired Student’s *t* test). The iliac diameter increased by 0.31 ± 0.02 mm before and 0.29 ± 0.07 mm after fMLP exposure (*P* = 0.7) and the diameter increased by 0.54 ± 0.11 mm before and 0.54 ± 0.09 mm after neuraminidase exposure (*P* = 0.98). In all cases, the shear stress changes before and after lumen exposure were not significantly different to each other.

**Conclusion:**

There was no significant reduction in flow mediated dilation of the iliac in response to any of the interventions conducted. Therefore, the vascular endothelial glycocalyx as whole is not required for flow mediated dilation in conduit arteries in the intact animal.

## Introduction

A recently published in vivo study has shown that individual removal of heparan sulfate and hyaluronan components the glycocalyx lining endothelial cells on the iliac did not significantly reduce vessel dilation in response to an increase in blood flow (Ruane-O’Hora et al., 2019). This indicates that the most abundant glycoproteins of the vascular glycocalyx may not be endothelial-blood flow mechanosensors in conduit vessels, as previously reported (Hecker et al., 1993; Mochizuki et al., 2003; Tarbell, Simon & Curry, 2014). In addition, immunohistochemistry also revealed that removal of heparan sulfate did not affect transmembrane syndecan expression, indicating that syndecans also do not play a part in nitric oxide flow mediated dilation in vivo, confirming data from a recent study (Bartosch, Mathews & Tarbell, 2017). However, it is possible that flow induced vasodilation was maintained since two constituents were removed separately; or, that the glycocalyx-mechanosensory role is a property of the entire structure and not just one or two of the many elements present.

Therefore, the purpose of the present study was to determine if concomitant removal of both heparan sulfate and hyaluronan enzymatically would affect flow-induced vasodilation. Further, N-Formylmethionine-leucyl-phenylalanine (fMLP) a chemotactic agent that degrades the glycocalyx in its entirety was also used. Finally, the role of glycocalyx-sialic acid residues in flow mediated dilation was also evaluated. All experiments were carried out an established vascular model using the iliac artery of the anesthetized pig (Markos et al., 2008).

## Materials and Methods

### Methods

Procedures were performed under license in accordance with Irish and European directive 2010/63/EU following approval by University College Cork animal research ethics committee and the Health Products Regulatory Authority (HPRA). Project authorization number: AE19130/P025.

#### Surgery and instrumentation

**Figure 1:**
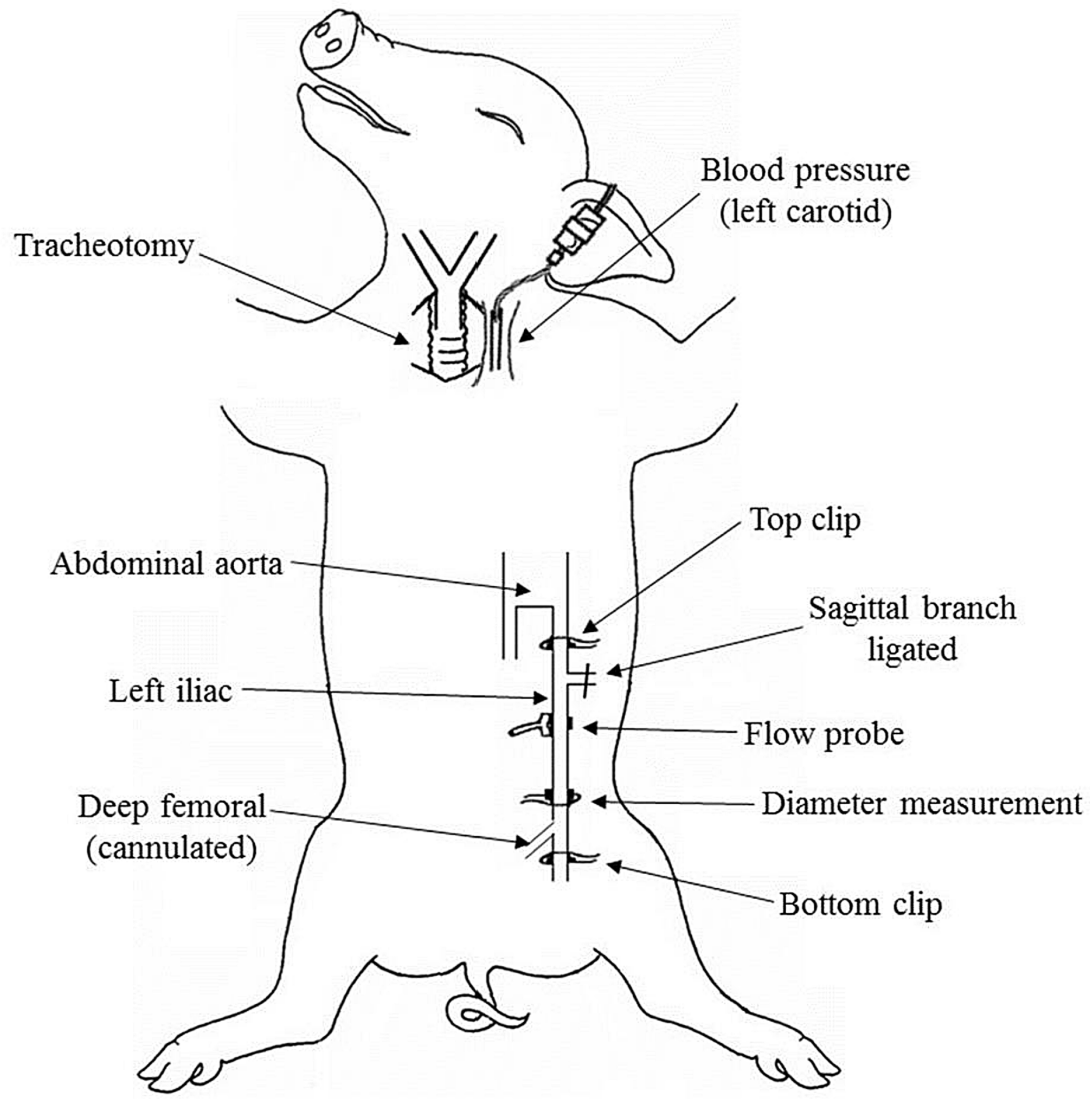
Schematic diagram showing the experimental preparation. Artists: Therese Ruane-O’Hora & Farouk Markos.

A total of 15 female landrace pigs (18.2–34.4 kg) were sedated with ketamine (14 mg kg^−1^) and xylazine (2.7 mg kg^−1^) i.m. A cannula was inserted into an ear vein and the animal was anesthetized with sodium pentobarbital (30 mg kg^−1^ for induction; 6 mg kg^−1^ h^−1^ for maintenance i.v.). Anesthetic infusion was maintained via a central line and an infusion pump (Harvard). Vital signs were monitored using SurgiVet AdvisorVital Signs Monitor (Smiths Medical, Dublin, OH, USA). An i-STAT analyzer (Abbot Point of Care Inc., Princeton, NJ, USA) was used to assess pH, P_a_CO_2_ and P_a_O_2_ and these parameters were kept within the normal range. A tracheotomy was performed and the pigs animals were ventilated with 40% O_2_ in room air using a ventilation pump (Harvard apparatus). A cannula was inserted into the carotid artery to measure systemic blood pressure (Grass; Grass Technologies, West Warwick, RI, USA). The left or right iliac artery was exposed and instrumented as previously described (Markos et al., 2008; Ruane-O’Hora et al., 2019), shown in Fig. 1. A cannula attached to a 3-way tap was placed in the deep femoral artery for injection of the compounds used to affect the glycocalyx-in-blood into the iliac. Piezoelectric crystals placed on opposite sides of the iliac measured diameter using a UDG sonomicrometer (Sonometrics Corporation, London, ON, Canada). A flow transducer (Transonic Systems Inc., Ithaca, NY, USA) was placed around the iliac for blood flow measurement. When needed, bulldog clips (top and bottom clips in Fig. 1) were placed above and below the diameter measurement site to isolate a test segment of iliac. All signals were recorded using a Power lab (AD Instruments Ltd., Oxford, UK) and a PC. Following experimental procedures animals were killed using a lethal injection of pentobarbitone and KCL i.v.

#### Protocol

**Figure 2:**
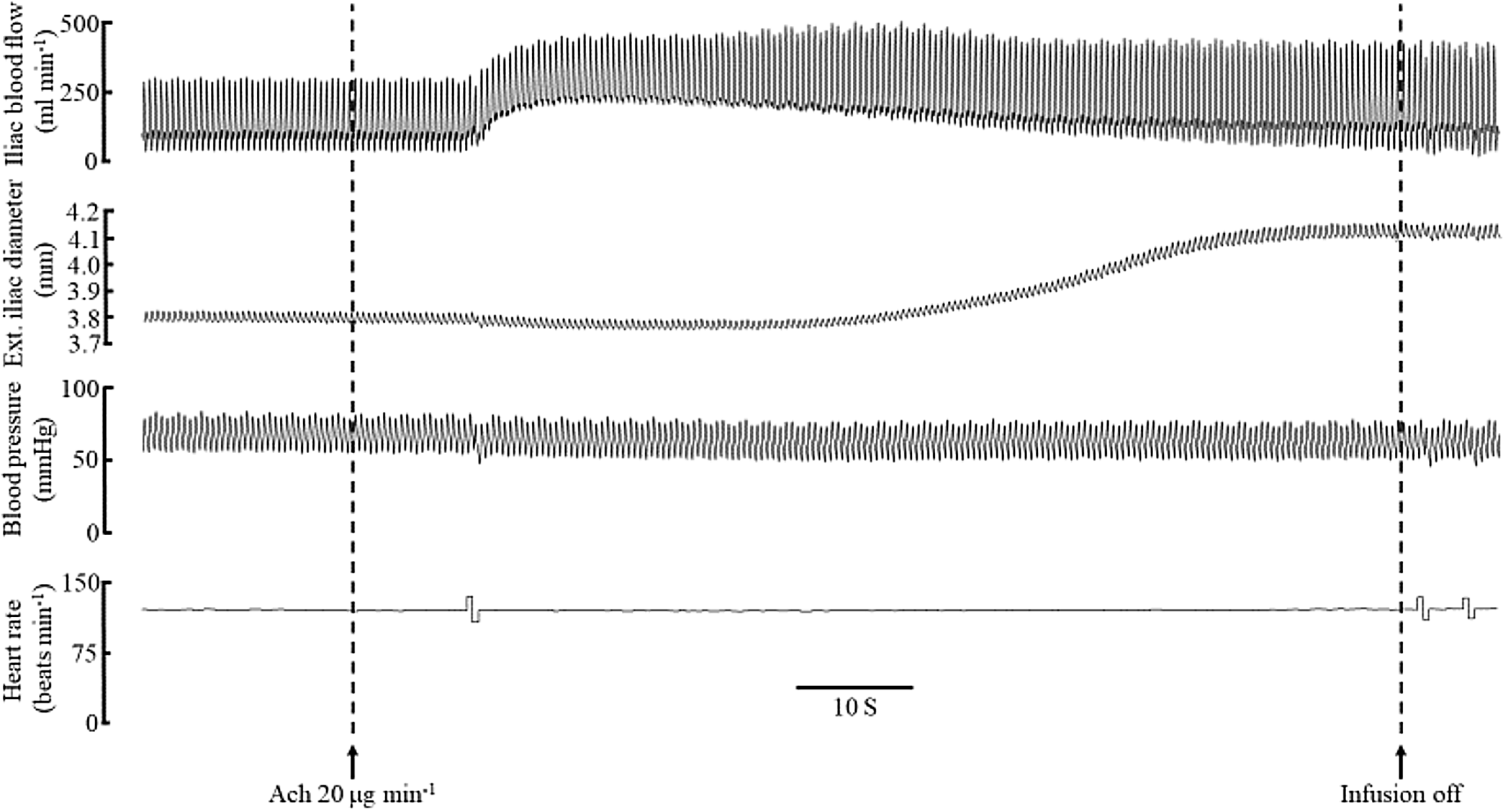
Records from pig Pig 21/14 showing a control flow mediated dilation of the iliac from a downstream infusion of acetylcholine (20 μg/min). The infusion was switched off soon after the peak diameter response, there was no effect of acetylcholine on systemic blood pressure (measured in the carotid) or heart rate.

Two acetylcholine infusions (5–20 μg/min) downstream of the iliac measurement site were carried out, one before (the control response) and one after a test segment was isolated and injected with arterial blood containing (a) heparinase III (100 mU ml^−1^) and hyaluronidase (14 μg ml^−1^); (b) neuraminidase (140 mU ml^−1^); (c) or fMLP (1 μM) for 20 min. An example of records obtained from one experiment (Pig 21/14) is shown in Fig. 2. Only one experimental intervention per iliac was conducted. It is important to note that acetylcholine infusion cannot directly interact with the diameter measurement site since it was infused downstream and also cannot circulate to affect systemic pressure (see Fig. 2) since acetylcholine is rapidly neutralized by blood cholinsterases.

#### Data analysis

Wall shear stress (N m^−2^) was calculated from the internal diameter (D), mean blood velocity (V_m_) and blood viscosity (μ = 4 mN s m^−2^). Shear stress (S) is given by: S = 8V_m_ · μ · D^−1^. Data was analyzed using paired Student’s *t* test, *P* < 0.05 was considered significant and data is presented as mean ± s.e.m.

#### Drugs used

Heparinase III, hyaluronidase, neuraminidase and fMLP were purchased from Sigma, anesthetics were purchased from Abbeyville Veterinary Hospital, Togher, Cork.

## Results

Baseline cardiovascular parameters at the onset of recording were as follows, heart rate averaged 111 ± 9 beats min^−1^ (range 55–197 beats min^−1^), mean arterial blood pressure was 75 ± 4 mm Hg (range 56–101 mm Hg), mean iliac blood flow was 120 ± 13 ml min^−1^ (range 58–220 ml min^−1^) and internal iliac diameter was 2 ± 0.012 mm (range 1.4–2.76 mm).

In Fig. 2, an example of records from one shows the effect of acetylcholine infusion downstream of the iliac diameter measurement site. The iliac test segment dilates in response to the increase in blood flow with little to no change in blood pressure or heart rate; since acetylcholine can only act in the vicinity and cannot re-circulate due to blood cholinesterases. This same protocol was repeated after the iliac segment was exposed to enzymes or fMLP.

### Heparinase III (100 mU ml^−1^) and hyaluronidase (14 µg ml^−1^)

**Figure 3:**
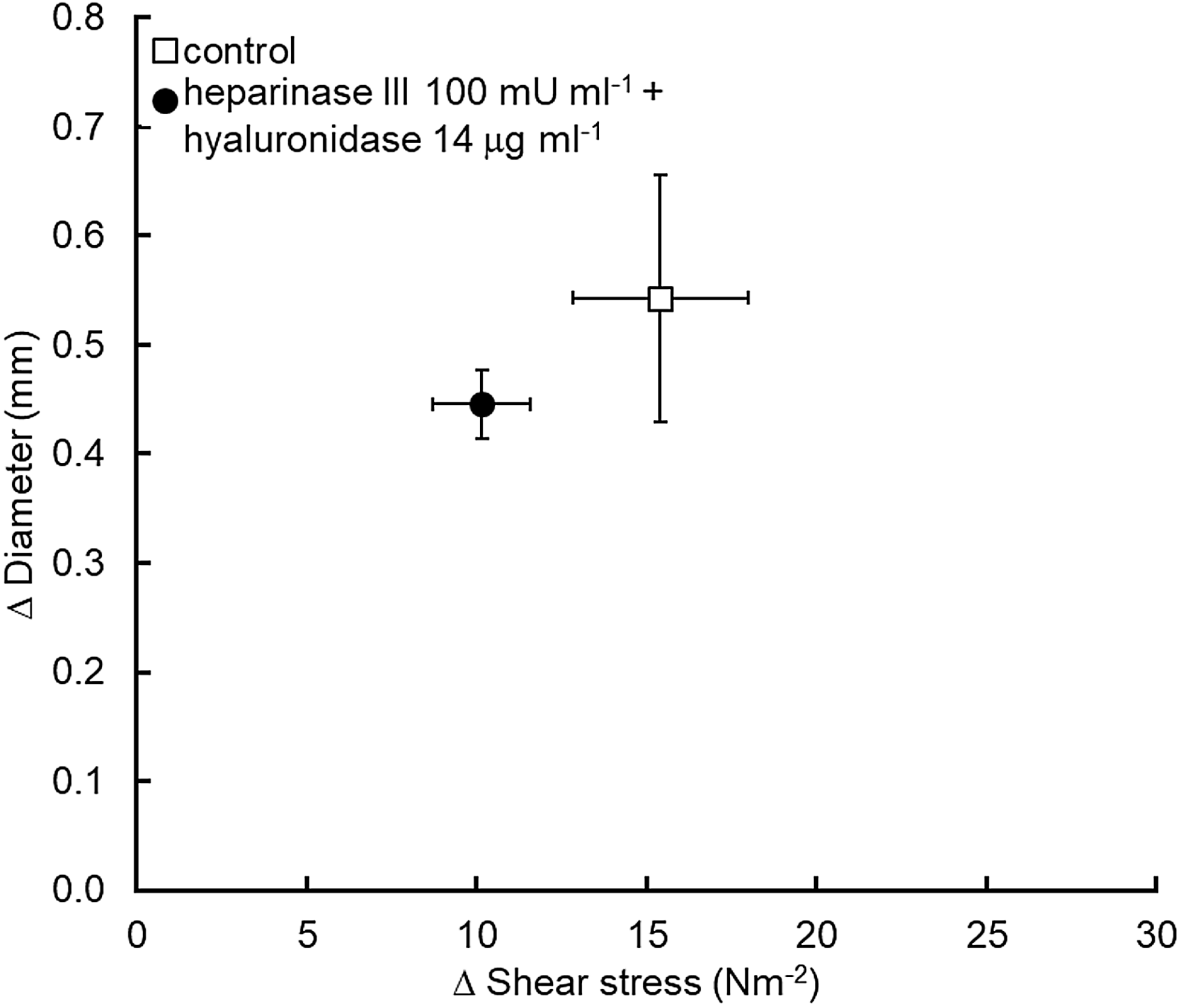
Concomitant application of both heparinase III and hyaluronidase did not cause a significant reduction in flow mediated dilation (*n* = 4 pigs).

Four pigs were used in this series of experiments and the data obtained is summarized in Fig. 3. Treatment of the iliac lumen for 20 min using both enzymes to selectively remove the heparan sulfate and hyaluronic acid components of the glycocalyx did not significantly reduce flow mediated iliac dilation, which increased by an average of 0.54 ± 0.11 mm before and 0.45 ± 0.03 mm after the enzymes (*P* = 0.42) for a statistically similar increase in shear stress; 15.4 ± 2.6 N m^−2^ for the control and 10.2 ± 1.4 N m^−2^ for the heparinase III + hyaluronidase experiment (*P* = 0.15).

### fMLP (1 µM)

**Figure 4:**
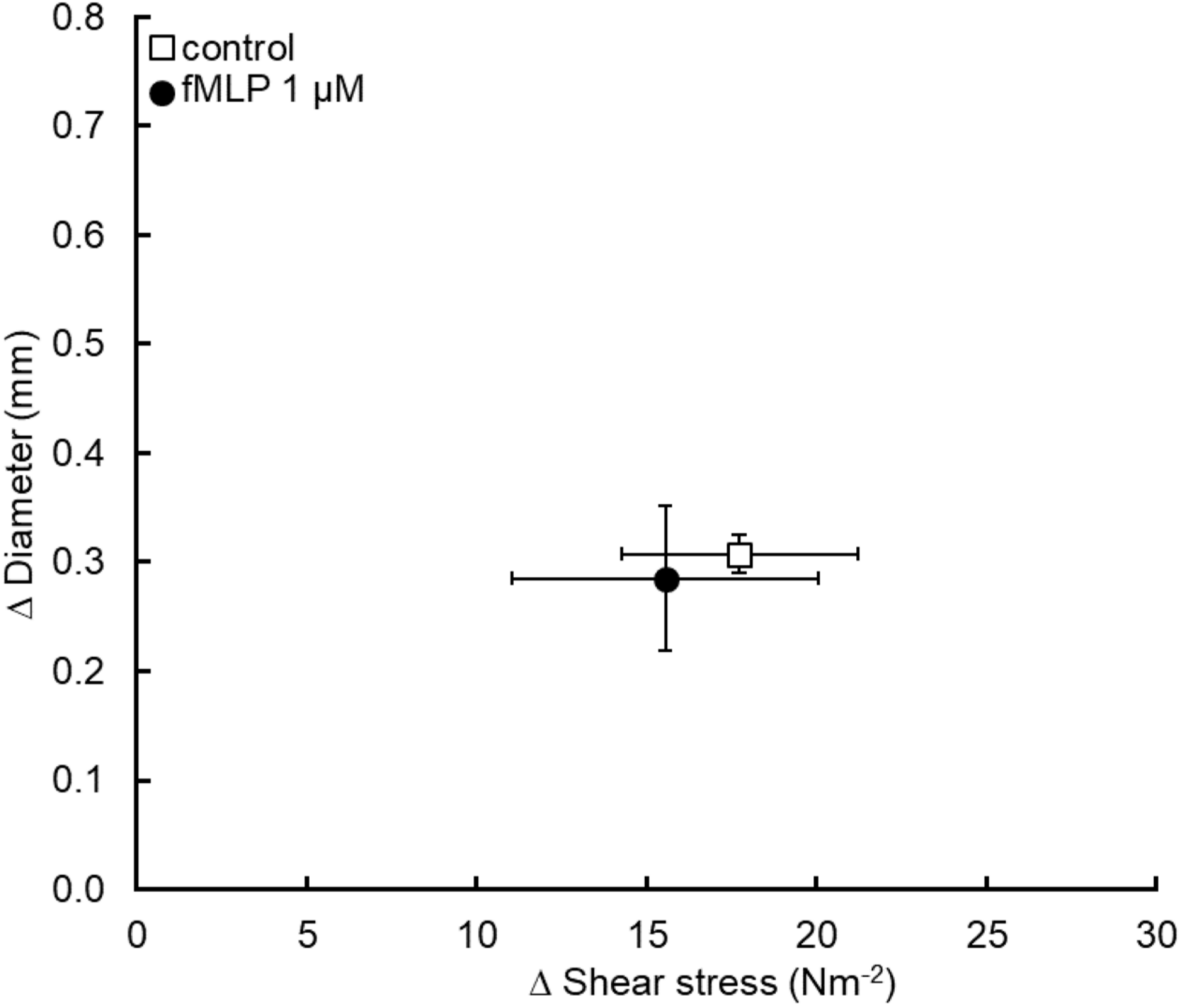
Application of fMLP to damage the entire glycocalyx in the iliac artery did not cause a significant reduction in flow mediated dilation (*n* = 6 pigs).

Six pigs were used and the data obtained is summarized in Fig. 4. Treatment of the iliac lumen for 20 min using the chemotactic agent fMLP to remove the entire glycocalyx also did not significantly reduce flow mediated dilation, diameter increased by 0.31 ± 0.02 mm before and 0.29 ± 0.07 mm after fMLP exposure (*P* = 0.7) for a statistically similar increase in shear stress; 17.7 ± 3.5 N m^−2^ for the control and 15.6 ± 4.5 N m^−2^ for the fMLP experiment (*P* = 0.68).

### Neuraminidase (140 mU ml^−1^)

**Figure 5:**
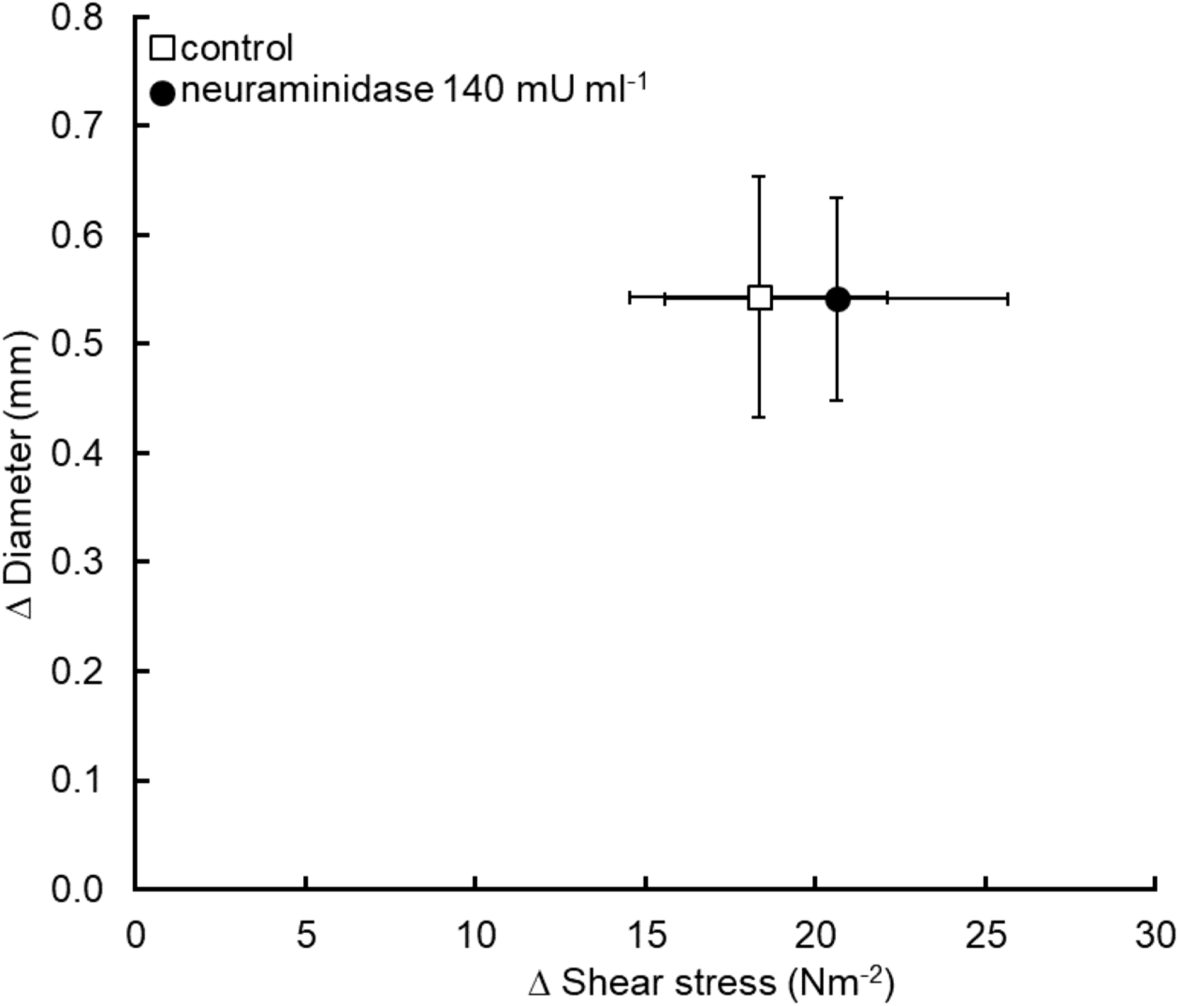
Removal of glycocalyx sialic acid residues using the enzyme neuraminidase did not cause a significant reduction in flow mediated dilation (*n* = 5 pigs).

Five pigs were used in this series of experiments and the data obtained is summarized in Fig. 5. Exposure of the iliac lumen for 20 min to neuraminidase to remove the sialic acid component of the glycocalyx did not significantly affect flow mediated dilation, diameter increased by 0.54 ± 0.11 mm before and 0.54 ± 0.09 mm after neuraminidase exposure (*P* = 0.98) for a statistically similar increase in shear stress; 18.3 ± 3.8 N m^−2^ for the control and 20.6 ± 5 N m^−2^ for the neuraminidase experiment (*P* = 0.6).

## Discussion

The main finding of the current study is that the entire glycocalyx structure in a conduit artery is not required to cause an increase diameter in response to an increase in blood flow, confirming and extending the conclusions of a recent aricle (Ruane-O’Hora et al., 2019). With regard to the effectiveness of the various compounds used to degrade the glycocalyx, Gao & Lipowsky (2010) previously showed that administration of a combination of enzymes caused much greater glycocalyx damage (reduction in thickness) than individual enzyme application. The concentration of enzymes used and the duration of exposure in the artery lumen was comparable to previous studies and was known to significantly reduce expression of both heparan sulfate and hyaluronic acid (Chappell et al., 2008; Florian et al., 2003; Ruane-O’Hora et al., 2019). The results post-neuraminidase treatment also show that sialic acid residues do not act as mediators of shear stress in vivo, which is at odds with findings from studies carried out in isolated artificially perfused arteries (Hecker et al., 1993; Kumagai, Lu & Kassab, 2009; Pohl et al., 1991).

fMLP, a known chemoattractant and inflammatory agent, was used to cause diffuse glycocalyx damage. While we do not have visual images to show degradation of the glycoclayx in response to fMLP in the present study, a previous investigation carried out in situ on exteriorized rat mesentery post capillary venules demonstrated a very rapid loss of glycocalyx after only 10 min (Mulivor & Lipowsky, 2004). Again, flow mediated dilation was not significantly affected following treatment of the artery lumen with fMLP. It should be noted that there is in vitro evidence that heparan sulfate bound glypican is involved in nitric oxide flow-mediated mechanotransduction (Ebong et al., 2014). Whether fMLP removed the glypicans together with the associated heparan sulfate is not clear.

There is obvious conflict between our data obtained in vivo and virtually all other studies in this field, which are conducted using either cultured cells or excised arteries perfused with physiological solutions. However, ex-vivo preparations are known to quickly lose glycocalyx components (Chappell et al., 2008), and cultured endothelial cells only express a rudimentary and incomplete glycocalyx (Chappell et al., 2008; Potter & Damiano, 2008). It is likely that glycocalyx-vascular function is artery specific, since glycocalyx damage in resistance arterioles due to maintained exposure to either hyaluronidase or fMLP causes a small but significant reduction in arterial conductance (Ruane-O’Hora & Markos, 2018).

## Conclusions

Overall, the findings support the importance of whole animal in vivo experiments and while conduit artery dilation is dependent on an intact endothelium it does not require a complete glycocalyx.

## Supplemental Information

10.7717/peerj.8725/supp-1 Supplemental Information 1 Raw data used to draw graphs, the stats & the baseline cardiovascular parameters.

## Additional Information and Declarations

### Data Availability

The following information was supplied regarding data availability: The raw data is available in the Supplemental File.
